# Healthy eating index 2015 might be associated with migraine headaches: Results from a Case–Control study

**DOI:** 10.1002/fsn3.4168

**Published:** 2024-04-21

**Authors:** Danial Fotros, Morvarid Noormohammadi, Mansoureh Togha, Zeinab Ghorbani, Azita Hekmatdoost, Pegah Rafiee, Zahra Torkan, Pedram Shirani, Hossein Ansari, Ahmadreza Karami, Faezeh Khorsha, Soodeh Razeghi Jahromi

**Affiliations:** ^1^ Department of Clinical Nutrition and Dietetics, Faculty of Nutrition and Food Technology Shahid Beheshti University of Medical Sciences Tehran Iran; ^2^ Department of Nutrition, School of Public Health Iran University of Medical Sciences Tehran Iran; ^3^ Student Research Committee, Faculty of Public Health Branch Iran University of Medical Sciences Tehran Iran; ^4^ Headache Department, Iranian Centre of Neurological Research, Neuroscience Institute Tehran University of Medical Sciences Tehran Iran; ^5^ Department of Clinical Nutrition School of Medicine Guilan University of Medical Sciences Rasht Iran; ^6^ Department of Neurology University of California san Diego (UCSD) San Diego California USA; ^7^ Multiple Sclerosis Research Center Neuroscience Institute Tehran University of Medical Sciences Tehran Iran

**Keywords:** case–control study, healthy eating index, migraine headaches

## Abstract

Migraine headaches are the most prevalent disabling primary headaches, affecting individuals at an active age. Dietary interventions are considered low‐cost and practical approaches to migraine prophylaxis. Hence, the present study aimed to assess the association between adherence to the Healthy Eating Index 2015 (HEI‐2015) and migraine headaches. The present case–control study was conducted on 476 newly diagnosed adults with migraine headaches, based on the International Classification of Headache Disorders 3rd edition (ICHDIII criteria(, and 512 healthy controls. Participants' dietary intakes were collected using a validated, 168‐item semi‐quantitative food frequency questionnaire (FFQ). The association between HEI‐2015 and migraine headaches was assessed using logistic regression models. Although the trend was not statistically significant, being in the 4th quantile of the HEI‐2015 was associated with about 50% lower odds of migraine headaches in both primary (adjusted for age and gender) (odds ratios (OR): 0.51, 95% confidence intervals (CI): 0.33, 0.78) and fully adjusted models (additionally adjusted for body mass index (BMI) and total calories) (adjusted OR: 0.50, 95%CI: 0.32, 0.77). Intriguingly, the odds of migraine headaches were significantly higher in those in the last quantile of “Total Fruits,” which is equal to more than 237 g per 1000 kcal (aOR: 2.96, 95%CI: 1.99, 4.41) and “Whole Fruits,” which is equal to more than 233 g per 1000 kcal (aOR: 2.90, 95%CI: 1.94, 4.31). Similarly, higher intakes of “Dairy,” which is equal to more than 138 g per 1000 kcal (aOR: 2.66, 95%CI: 1.71, 4.14), and “Total Protein Foods,” which is equal to more than 259 g per 1000 kcal (aOR: 2.41, 95%CI: 1.58, 3.70), were associated with higher odds of migraine headaches. The current study revealed an indirect association between HEI‐2015 and its components, including “Greens and Beans,” “Whole Grains,” “Refined Grains,” and “Added Sugars” and lower odds of migraine headaches.

## Graphical abstract

This case–control study showed that moderate adherence to the HEI‐2015 (HEI‐2015 scores ranging from 65 to 76) reduces the odds of migraine headaches substantially. Additionally, consumption of “Total Vegetables,” “Greens and Beans,” and “Whole Grains” is associated with lower odds of migraine headaches. Alternatively, moderate fruit consumption has been found to lower migraine headache odds, while consuming too much fruit is associated with higher migraine headache odds.

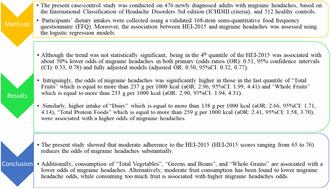

## 1 INTRODUCTION

Migraine is the most common disabling primary headache, and about 12% of individuals worldwide are affected by migraine headaches (Goadsby & Holland, 2019; Ruschel & De Jesus, 2021; Stovner et al., 2022). A majority of migraine sufferers are between the ages of 15 and 49, peaking between 35 and 39, considered as active age (Reuter, 2018). Migraine headaches account for 42.1 million Years Lived with Disability (YLDs), which was reported to be responsible for 4.8% of overall YLDs in 2019 (Irimia et al., 2022). Upon removing certain stimulants or exposing oneself to certain factors, migraine headaches may progress (Martin & Behbehani, 2001). The most prevalent trigger factors have been reported to be stress, hormone alterations in women, hunger, climate change, alterations in sleep pattern, perfume or odor, neck pain, light(s), alcohol, smoking, heat, some food items, exercise, and sexual activity (Kelman, 2007). Several of these factors were proven to contribute, whereas others can only be regarded as potential factors (Burstein et al., 2015). Migraine headaches are caused by varying intracranial and extracranial alterations caused by multiple primary neuronal dysfunctions (Burstein et al., 2015).

Migraine treatment involves modifying or avoiding migraine triggers, as well as taking medication for symptom relief or prophylaxis (Steiner et al., 2019). On the economic side, migraine overall health expenditures in Europe are estimated to be between €50 billion and €111 billion, comprising both direct (7%) and indirect (93%) healthcare expenditures (Ashina et al., 2021). Even though dietary interventions can be considered a low‐cost and practical approach to migraine prophylaxis, reducing migraine costs as a result, they have received relatively little study in the past. A new concept in nutrition epidemiology is the diet quality, which assesses the synergistic effects of food instead of the independent effects of one nutrient. One approach for assessing diet quality is the Healthy Eating Index (HEI) (Krebs‐Smith et al., 2018). To assess diet quality through HEI, food items including “Total Vegetables,” “Greens and Beans,” “Whole Grains,” “Seafood and Plant Protein,” “Refined Grains,” “Total Fruits,” “Whole Fruits,” “Dairy,” “Total Protein Foods,” “Added Sugars,” “Saturated Fats (SFA),” and “Poly Unsaturated Fat (PUFA)+ Mono Unsaturated Fat (MUFA) to SFA” ratio components were calculated. Scores on the HEI‐2015 range from 0 to 100, and higher scores reflect better diet quality. An indirect association is reported between HEI‐2015 and inflammatory status (Millar et al., 2021). However, to the best of our knowledge, no research has been conducted on the relationship between HEI‐2015 and the odds of migraine headaches. Therefore, the current study aimed to investigate the potential correlation between adherence to the HEI‐2015 dietary guidelines and the odds of migraine headaches.

## 2 MATERIALS AND METHODS

### 2.1 Ethical considerations

The informed consent form was required for all participants prior to enrollment. The vice‐chancellor of research affairs’ ethics committee reviewed and approved the study methods and design (Research Ethics Committees of the Neuroscience Institute; ethics code: IR.TUMS.NI.REC.1398.010, approval date: 08/08/2019). The latest version of the Helsinki Declaration was followed throughout the entire procedure.

### 2.2 Study design and participants

The current case–control study was performed on newly diagnosed patients with migraine headaches (maximum 1 month after the diagnosis) recruited from adults referred to the tertiary headache clinic of Sina University Hospital and a private headache clinic in Tehran, Iran, between 2015 and 2018. Healthy volunteer controls were selected from the general population. Individual recruitment was based on a convenience sampling approach. Detailed information for the research protocol has been published before (Razeghi Jahromi et al., 2019). The diagnosis approach for migraine headaches was based on the International Classification of Headache Disorders, 3rd edition (ICHDIII) (Olesen, 2018), conducted by an expert headache‐specialist neurologist. All individuals are older than 18 years. Those with a history of chronic diseases, dietary‐related chronic metabolic disorders, and any neurological conditions other than migraine, pregnant and lactating women, and those adhering to a specific dietary pattern during the previous year were not included in this study. The analysis did not include individuals who consumed less than 600 or more than 4200 kcal or had a BMI less than 18.5 or more than 34.9 kg/m^2^. Further information is provided in detail in the previous study (Razeghi Jahromi et al., 2019).

### 2.3 Assessment of anthropometric data

The anthropometric measures were assessed by two qualified nutritionists. Using a Seca medical scale (with an accuracy of 0.5 kg; Seca GmbH & Co. KG, Hamburg, Germany) and a standard stadiometer (with an accuracy of 0.1 cm), participants were measured for their body weight and height. The body mass index (BMI) is calculated by dividing the weight in kilograms by the square of the height in meters.

### 2.4 Dietary intake

A validated 168‐item semi‐quantitative food frequency questionnaire (FFQ) was used to evaluate participants' regular food consumption during the previous year prior to the diagnosis of migraine headaches and during the last year prior to the interview in the healthy controls (Esfahani et al., 2010). Face‐to‐face interviews were conducted by trained interviewers blinded to the study group. The food album was used to estimate serving size based on individuals' reported intake frequency (daily, weekly, monthly, or yearly) for each food item. Then, using household measures, reported frequencies were converted to grams per day (Ghaffarpour et al., 1999). Daily energy and nutrient intake were computed using the US Department of Agriculture food composition database, modified for Iranian foods (Aminianfar et al., 2020). Due to the lack of some food items in the USDA food composition database, the Iranian food composition database was applied alternatively for these food items (Azar & Sarkisian, 1980).

The HEI‐2015 is an approach to assessing diet quality. It is designed based on the 2015–2020 Dietary Guidelines for Americans and includes 13 components as provided in Table S1 (Krebs‐Smith et al., 2018). To calculate HEI‐2015, FFQ food items were converted to cup and ounce equivalents. The “Added Sugars” and “Saturated Fats” components were calculated as percentages of total calorie intake. Other components except for “Fatty Acids” were calculated as the consumption in cups/ounces per 1000 kcal. The maximum and minimum scores were calculated as shown in Table S1. The HEI‐2015 score ranged from 0 to100.

### 2.5 Statistical analysis

The statistical analysis was performed using SPSS software version 26. A Kolmogorov–Smirnov test and a Q–Q plot were used to assess the normality of quantitative values. The quantitative and qualitative values are expressed as median (interquartile range (IQR)) and frequency (percentages), respectively. The Chi‐square test and the Student's *t*‐test were used to compare qualitative and quantitative values, respectively. Furthermore, the Mann–Whitney test was used for non‐normally distributed quantitative values. To analyze the association between HEI‐2015 and migraine headaches, logistic regression was used to calculate primary and fully adjusted odds ratios (ORs) with 95% confidence intervals (CIs). The primary adjusted ORs were adjusted for age and gender, while the fully adjusted ORs were additionally adjusted for BMI (kg/m^2^) and total calorie intake (kcal/day). All hypothesis tests were assumed to be statistically significant if the *p*‐value was less than .05.

## 3 RESULTS

A total of 514 migraine headache patients and 582 healthy controls were enrolled. Nevertheless, 38 migraine headache patients and 70 healthy controls were excluded from the study due to consuming too many calories (>4200 kcal) or having a BMI of less than 18.5 kg/m2 or greater than 34.9 kg/m2. Finally, 476 patients with migraine headaches (93.9% were women) and 512 healthy controls (94.7% were women) were included in the present study. The general characteristics and dietary intakes of cases and controls are presented in Table 1. Patients with migraine headaches were younger and had a lower BMI (*p*‐value <.001). Besides, the consumption of total animal‐based protein intake (g/day), total fat intake (g/day), total animal‐based fat intake (g/day), total cholesterol intake (mg/day) (*p*‐value <.001), and total plant‐based fat intake (g/day) (*p*‐value = .048) were significantly higher in the case group. In addition, the consumption of total plant‐based protein intake (g/day) (*p*‐value <.001) and total carbohydrate intake (g/day) (*p*‐value = .010) was higher in healthy controls.

**TABLE 1:** General characteristics of patients with migraine headaches and healthy controls.

Characteristics^a^	Healthy controls *N* = 512	Patients with migraine headaches *N* = 476	*p* Value^b^
Sex, Female, number (percentage)	485 (94.7)	447 (93.9)	.578
Age, year, median (Q_1_–Q_3_)	44.00 (33.00, 54.75)	35.50 (30.00, 43.00)	<.001*
BMI	27.03 (24.77, 29.75)	25.37 (22.55, 28.63)	<.001*
Total calories intake (kcal/day)	2112.73 (1796.82, 2483.10)	2151.59 (1635.47, 2606.07)	.768
Total protein intake (g/day)	89.81 (72.68, 105.19)	86.52 (71.02, 104.38)	.230
Total plant‐based protein intake (g/day)	38.50 (30.44, 46.68)	30.82 (24.03, 40.54)	<.001*
Total animal‐based protein intake (g/day)	34.70 (26.33, 44.76)	40.52 (32.28, 51.70)	<.001*
Total fat intake (g/day)	77.80 (62.83, 101.52)	90.88 (65.09, 123.25)	<.001*
Total plant‐based fat intake (g/day)	53.95 (41.62, 70.55)	56.01 (41.18, 81.91)	.048*
Total animal‐based fat intake (g/day)	23.56 (17.58, 31.82)	28.75 (21.81, 42.88)	<.001*
Total carbohydrates intake (g/day)	227.24 (189.66, 273.54)	211.18 (155.29, 293.71)	.010*
Total cholesterol intake (mg/day)	176.18 (130.88, 237.52)	223.29 (172.24, 294.10)	<.001*

Abbreviation: BMI, body mass index.

^a^ Values are median (interquartile range IQR) unless otherwise noted.

^b^ Using the Kruskal–Wallis test.

*statistically significant.

HEI‐2015 components (g/day per 1000 kcal) are represented in Table 2. The total HEI‐2015 score was significantly higher in healthy controls (*p*‐value <.001), and according to the HEI‐2015 components, consumption of “Total Vegetables,” “Greens and Beans,” “Whole Grains,” “Seafood and Plant Protein,” and “Refined Grains,” and PUFA+MUFA to SFA ratio were significantly higher in healthy controls (*p*‐value <.001). Patients with migraine headaches consumed significantly higher “Total Fruits,” “Whole Fruits,” “Dairy,” “Total Protein Foods,” and “Saturated Fats” (*p*‐value <.001) (Table 2).

**TABLE 2:** Healthy eating index 2015 and components separated by patients with migraine headaches and healthy controls.

Healthy eating index 2015 and components^a^	Healthy controls, *N* = 512	Patients with migraine headaches, *N* = 476	*p* Value^b^
Total Fruits (g per 1000 kcal)	159.62 (111.57, 221.77)	222.08 (108.21, 432.55)	<.001*
Whole Fruits (g per 1000 kcal)	158.27 (110.00, 217.80)	201.30 (104.89, 429.14)	<.001*
Total Vegetables (g per 1000 kcal)	154.28 (119.25, 215.79)	138.06 (80.01, 210.57)	<.001*
Greens and Beans (g per 1000 kcal)	51.09 (34.42, 68.13)	34.23 (20.36, 60.63)	<.001*
Whole grains (g per 1000 kcal)	16.39 (7.30, 36.82)	9.98 (3.68, 25.93)	<.001*
Dairy (g per 1000 kcal)	120.09 (78.91, 167.51)	146.69 (101.38, 193.38)	<.001*
Total Protein Foods (g per 1000 kcal)	189.17 (143.62, 242.57)	213.05 (163.92, 288.42)	<.001*
Seafood and Plant Protein (g per 1000 kcal)	30.36 (20.39, 43.82)	22.35 (15.00, 35.05)	<.001*
Fatty Acids ((PUFA+MUFA)/SFA)	2.38 (1.98, 2.88)	1.94 (1.54, 2.56)	<.001*
Refined Grains (g per 1000 kcal)	156.69 (110.65, 201.42)	119.35 (78.48, 162.62)	<.001*
Sodium (g per 1000 kcal)	0.68 (0.51, 0.91)	0.68 (0.56, 0.86)	.415
Added Sugars (g per 1000 kcal)	29.46 (17.84, 45.08)	25.55 (14.86, 47.07)	.107
Saturated Fats (g per 1000 kcal)	9.44 (7.85, 11.09)	11.10 (8.77, 14.24)	<.001*
The total healthy eating index score	71 (66.25, 75)	69 (61, 75)	<.001*

^a^ Values are median (interquartile range IQR) unless otherwise noted.

^b^ Mann–Whitney *U* test.

*Statistically significant.

In the primary model adjusted for age (years) and gender (male/female), although the trend was not statistically significant (*p*‐value = .169), patients in the 2nd, 3rd, and 4th quantiles of the HEI‐2015 score had 49%–58% lower odds of migraine headaches, which was statistically significant (OR (95%CI): 0.42 (0.28, 0.61); OR (95%CI): 0.51 (0.33, 0.78); and OR (95%CI): 0.51 (0.33, 0.78)). Similarly, in the fully adjusted model, additionally adjusted for BMI (kg/m^2^) and total calories (kcal), the odds of migraine headaches were 49%–59% lower for the patients in the 2nd, 3rd, and 4th quantiles of the HEI‐2015 (aOR: 0.50, 95%CI: 0.32, 0.77). According to both primary and fully adjusted models, patients who were in the 2nd, 3rd, and 4th quantiles of “Total Fruits,” which is equal to 103–237 grams per 1000 kcal, were significantly less likely to suffer migraine headaches (aOR: 0.48, 95%CI: 0.30, 0.77, P_trend_ < 0.001), and “Whole Fruits,” which is equal to 101–233 g per 1000 kcal (aOR: 0.44, 95%CI: 0.27, 0.72, P_trend_ < 0.001), but was significantly higher in those in the last quantile of “Total Fruits,” which is equal to more than 237 g per 1000 kcal (aOR: 2.96, 95%CI: 1.99, 4.41) and “Whole Fruits,” which is equal to more than 233 g per 1000 kcal (aOR: 2.90, 95%CI: 1.94, 4.31, P_trend_ < 0.001). In the primary or fully adjusted model, “Total Vegetables” intake was associated with lower odds of migraine headaches (aOR: 0.66, 95%CI: 0.44, 0.99); however, the trend was not statistically significant. In both primary and adjusted models, an inverse association was observed between migraine headaches and intake of “Greens and Beans” (aOR: 0.32, 95%CI: 0.21, 0.48, P_trend_ < 0.001), “Whole Grains” (aOR: 0.38, 95%CI: 0.24, 0.59, P_trend_ < 0.001), “Seafood and Plant Protein” (aOR: 0.34, 95%CI: 0.22, 0.52, P_trend_ < 0.001), (PUFA+MUFA)/SFA ratio (aOR: 0.28, 95%CI: 0.18, 0.44, P_trend_ < 0.001), “Refined Grains” (aOR: 0.14, 95%CI: 0.08, 0.23, P_trend_ < 0.001), and “Added Sugars” (aOR: 0.54, 95%CI: 0.35, 0.84, P_trend_ < 0.001). On the other hand, higher intakes of “Dairy,” which is equal to more than 138 g per 1000 kcal (aOR: 2.66, 95%CI: 1.71, 4.14, P_trend_ < 0.001), “Total Protein Foods,” which is equal to more than 259 g per 1000 kcal (aOR: 2.41, 95%CI: 1.58, 3.70, P_trend_ < 0.001), and “Saturated Fats,” which is equal to more than 12 g per 1000 kcal (aOR: 2.94, 95%CI: 1.91, 4.53, P_trend_ < 0.001), were associated with higher odds of migraine headaches in both models. In addition, those in the 2nd, 3rd, and 4th quantiles of the “Sodium” intake had higher odds of migraine headaches (aOR: 1.68, 95%CI: 1.06, 2.66), in both primary and fully adjusted models (Table 3).

**TABLE 3:** Odds ratio (OR) estimates and 95% confidence intervals (CIs) for the association between the healthy eating index 2015 and components and migraine headaches in a sample of patients with migraine headaches compared to healthy individuals based on the tertiles of healthy eating index 2015.^a^

Healthy eating index 2015 and components	Tertiles of healthy eating index 2015					P_trend_
	1st	2nd	3rd	4rd	5rd	
Healthy eating index 2015	<65	65–70	70–73	73–76	76 <	
No. Cases/No. Controls	184/109	82/125	61/90	61/93	88/95	
Primary Model^b^	1.00 (Ref.)	0.42 (0.28, 0.61)	0.50 (0.33, 0.77)	0.51 (0.33, 0.78)	0.77 (0.52, 1.15)	0.169
Model 1^c^	1.00 (Ref.)	0.41 (0.28, 0.61)	0.51 (0.33, 0.79)	0.50 (0.32, 0.77)	0.77 (0.51, 1.16)	0.209
Total Fruits (g per 1000 kcal)	<103	103–141	141–181	181–237	237 <	
No. Cases/No. Controls	110/102	55/103	40/102	51/103	220/102	
Primary Model^b^	1.00 (Ref.)	0.49 (0.31, 0.77)	0.34 (0.21, 0.55)	0.47 (0.29, 0.74)	2.81 (1.90, 4.16)	<0.001*
Model 1^c^	1.00 (Ref.)	0.49 (0.31, 0.78)	0.33 (0.20, 0.54)	0.48 (0.30, 0.77)	2.96 (1.99, 4.41)	<0.001*
Whole Fruits (g per 1000 kcal)	<101	101–139	139–178	178–233	233 <	
No. Cases/No. Controls	112/102	59/103	46/102	45/103	214/102	
Primary Model^b^	1.00 (Ref.)	0.52 (0.33, 0.81)	0.41 (0.26, 0.66)	0.43 (0.27, 0.69)	2.76 (1.86, 4.09)	<0.001*
Model 1^c^	1.00 (Ref.)	0.52 (0.33, 0.82)	0.40 (0.25, 0.65)	0.44 (0.27, 0.72)	2.90 (1.94, 4.31)	<0.001*
Total Vegetables (g per 1000 kcal)						
	<110	110–139	139–180	180–229	229 <	
No. Cases/No. Controls	189/102	50/103	64/102	78/103	95/102	
Primary Model^b^	1.00 (Ref.)	0.29 (0.19, 0.45)	0.37 (0.24, 0.55)	0.54 (0.36, 0.81)	0.65 (0.44, 0.96)	0.080
Model 1^c^	1.00 (Ref.)	0.30 (0.19, 0.46)	0.37 (0.24, 0.56)	0.57 (0.38, 0.86)	0.66 (0.44, 0.99)	0.137
Greens and Beans (g per 1000 kcal)	<32	32–43	43–57	57–76	76 <	
No. Cases/No. Controls	214/102	87/103	47/102	50/103	78/102	
Primary Model^b^	1.00 (Ref.)	0.43 (0.29, 0.64)	0.22 (0.14, 0.35)	0.21 (0.14, 0.33)	0.37 (0.24, 0.55)	<0.001*
Model 1^c^	1.00 (Ref.)	0.40 (0.27, 0.60)	0.20 (0.13, 0.31)	0.19 (0.12, 0.29)	0.32 (0.21, 0.48)	<0.001*
Whole grains (g per 1000 kcal)						
	<6	6–11	11–23	23–44	44 <	
No. Cases/No. Controls	166/102	91/103	83/102	76/103	60/102	
Primary Model^b^	1.00 (Ref.)	0.53 (0.36, 0.79)	0.44 (0.29, 0.65)	0.48 (0.32, 0.72)	0.37 (0.24, 0.57)	<0.001*
Model 1^c^	1.00 (Ref.)	0.54 (0.36, 0.80)	0.45 (0.30, 0.67)	0.48 (0.32, 0.73)	0.38 (0.24, 0.59)	<0.001*
Dairy (g per 1000 kcal)						
	<69	69–103	103–138	138–186	186 <	
No. Cases/No. Controls	59/102	63/103	98/102	117/103	139/102	
Primary Model ^b^	1.00 (Ref.)	1.04 (0.65, 1.67)	1.55 (0.99, 2.43)	1.93 (1.24, 3.00)	2.51 (1.62, 3.87)	<0.001*
Model 1^c^	1.00 (Ref.)	1.07 (0.66, 1.73)	1.52 (0.96, 2.39)	2.01 (1.28, 3.14)	2.66 (1.71, 4.14)	<0.001*
Total Protein Foods (g per 1000 kcal)						
	<135	135–173	173–205	205–259	259 <	
No. Cases/No. Controls	75/102	58/103	82/102	101/103	160/102	
Primary Model^b^	1.00 (Ref.)	0.78 (0.49, 1.25)	1.08 (0.69, 1.68)	1.31 (0.85, 2.01)	2.24 (1.48, 3.38)	<0.001*
Model 1^c^	1.00 (Ref.)	0.77 (0.48, 1.23)	1.07 (0.69, 1.68)	1.36 (0.88, 2.11)	2.41 (1.58, 3.70)	<0.001*
Seafood and Plant Protein (g per 1000 kcal)						
	<19	19–27	27–34	34–46	46 <	
No. Cases/No. Controls	184/102	104/103	59/102	57/103	72/102	
Primary Model^b^	1.00 (Ref.)	0.51 (0.35, 0.76)	0.29 (0.19, 0.44)	0.28 (0.18, 0.44)	0.35 (0.23, 0.54)	<0.001*
Model 1^c^	1.00 (Ref.)	0.50 (0.33, 0.74)	0.28 (0.18, 0.43)	0.27 (0.17, 0.42)	0.34 (0.22, 0.52)	<0.001*
Fatty Acids ((PUFA+MUFA)/SFA)	<1.89	1.89–2.24	2.24–2.55	2.55–3.00	3.00 <	
No. Cases/No. Controls	226/102	96/103	34/102	72/103	48/102	
Primary Model^b^	1.00 (Ref.)	0.46 (0.31, 0.67)	0.17 (0.11, 0.27)	0.37 (0.25, 0.56)	0.28 (0.18, 0.44)	<0.001*
Model 1^c^	1.00 (Ref.)	0.47 (0.32, 0.69)	0.17 (0.11, 0.28)	0.38 (0.25, 0.57)	0.28 (0.18, 0.44)	<0.001*
Refined Grains (g per 1000 kcal)						
	<98	98–135	135–176	176–214	214 <	
No. Cases/No. Controls	170/102	124/103	100/102	46/103	36/102	
Primary Model^b^	1.00 (Ref.)	0.58 (0.39, 0.86)	0.42 (0.28, 0.63)	0.20 (0.12, 0.32)	0.15 (0.09, 0.24)	<0.001*
Model 1^c^	1.00 (Ref.)	0.55 (0.37, 0.82)	0.42 (0.28, 0.63)	0.18 (0.11, 0.30)	0.14 (0.08, 0.23)	<0.001*
Sodium (mg per 1000 kcal)						
	<469	469–612	612–736	736–974	974 <	
No. Cases/No. Controls	53/102	117/103	110/102	117/103	79/102	
Primary Model^b^	1.00 (Ref.)	1.92 (1.23, 3.01)	1.74 (1.11, 2.74)	1.63 (1.04, 2.57)	1.07 (0.67, 1.71)	0.649
Model 1^c^	1.00 (Ref.)	1.96 (1.24, 3.08)	1.79 (1.13, 2.84)	1.68 (1.06, 2.66)	1.08 (0.67, 1.75)	0697
Added Sugars (g per 1000 kcal)						
	<15	15–25	25–35	35–51	51 <	
No. Cases/No. Controls	121/102	116/103	55/102	78/103	106/102	
Primary Model^b^	1.00 (Ref.)	0.75 (0.50, 1.12)	0.31 (0.19, 0.49)	0.37 (0.24, 0.57)	0.53 (0.35, 0.80)	<0.001*
Model 1^c^	1.00 (Ref.)	0.77 (0.51, 1.16)	0.31 (0.20, 0.50)	0.37 (0.24, 0.57)	0.54 (0.35, 0.84)	<0.001*
Saturated Fats (g per 1000 kcal)						
	<7	7–9	9–10	10–12	12 <	
No. Cases/No. Controls	55/102	70/103	65/102	79/103	207/102	
Primary Model^b^	1.00 (Ref.)	1.08 (0.68, 1.74)	0.97 (0.60, 1.57)	1.11 (0.70, 1.78)	2.99 (1.95, 4.58)	<0.001*
Model 1^c^	1.00 (Ref.)	1.11 (0.69, 1.80)	0.97 (0.60, 1.57)	1.10 (0.69, 1.76)	2.94 (1.91, 4.53)	<0.001*

Abbreviations: MUFA, monounsaturated fatty acid; PUFA, polyunsaturated fatty acid; SFA, saturated fatty acid.

*Statistically significant.

^a^ Logistic regression model.

^b^ Adjusted for age (years) and gender (male/female).

^c^ Additionally adjusted for BMI (kg/m^2^) and total calories (kcal).

## 4 DISCUSSION

Based on our knowledge, the present study is the first to investigate the association between HEI‐2015 and migraine headaches. In an overall view to diet quality using HEI‐2015, being in the 2nd, 3rd, and 4th quantiles of HEI‐2015 is associated with substantially lower odds of migraine headaches. Dietary intakes of “Total Vegetables,” “Greens and Beans,” “Whole Grains,” and “Seafood and Plant Protein,” PUFA and MUFA compared to SFA, “Refined Grains,” and even “Added Sugars” seem to have protective effects against migraine headaches, as shown by the current study. Besides, this study reveals the deleterious effects of high intakes of “Dairy” and “Total Protein Foods,” which include red meat and animal protein foods, “Saturated Fats,” and “Sodium” on migraine headaches. Moderate intakes of “Dairy,” “Total Protein Foods,” and “Saturated Fats” do not show this direct association. It is shown that moderate intake of “Total Fruits,” which includes fruits and natural fruit juices, and “Whole Fruits,” which includes all kind of fruits, has protective effects against migraine headaches. Nevertheless, higher consumption of “Whole Fruits” and “Total Fruits” may be associated with higher migraine odds.

In a similar study in 2015, Evans et al. showed no substantial difference between dietary intakes in healthy participants and patients with migraine headaches, but found an inverse association between the HEI‐2005 score and migraine headaches among normal‐weight women (Evans et al., 2015). However, in the mentioned study, dietary intakes were collected using 24 h dietary recall, and the 2005 version of the HEI was calculated, which are two major differences from the current study (Evans et al., 2015). HEI is in part similar to dietary approaches to stop hypertension (DASH) and Mediterranean dietary patterns regarding emphasis on fruits, vegetables, and legume intake (Davis et al., 2015; Steinberg et al., 2017). Higher DASH diet adherence has been shown to be associated with lower headache severity (Bakırhan et al., 2021; Mirzababaei et al., 2020) and a lower mean headache duration for each attack (Mirzababaei et al., 2020). Additionally, the Mediterranean diet has been shown to provide protection against headache frequency, headache duration, migraine headache index score, and headache impact test‐6 (HIT‐6) (Arab et al., 2021). Patients with low Mediterranean diet adherence have been shown to have a higher rate of migraine‐related severe disability (assessed by the Migraine Disability Assessment Scale (MIDAS)) and more severe and frequent attacks (Bakırhan et al., 2021). Another study found that the combination of the Mediterranean diet and dietary approaches to stop hypertension, the MIND diet, was negatively associated with migraine intensity, frequency, and duration (Askarpour et al., 2020). In a clinical trial involving 42 patients with migraine, the administration of a low‐fat vegan diet followed by an elimination diet for 12 weeks resulted in substantial pain reduction when compared to consuming a placebo diet (Bunner et al., 2014).

In the present study, either vegetable and greens and beans consumption or moderate fruit consumption is shown to prevent migraine headaches, which is in agreement with our previous study regarding fruits and vegetable consumption in children. (Ariyanfar et al., 2019). Similarly, in other studies, fruit and vegetable consumption was substantially higher in healthy patients than in patients with migraine headaches (Nazari & Eghbali, 2012) and was indirectly associated with primary headaches among Iranian university students (Mansouri et al., 2021). Besides, higher intakes of vegetables, fruits, and legumes were also associated with a decreased severity of migraine attacks (Bakırhan et al., 2021).

The main probable mechanism by which dietary patterns may provide protective effects regarding migraine headaches is related to their anti‐inflammatory and anti‐oxidative properties (Estruch, 2010; Visioli & Galli, 2001). Neuroinflammation, and consequently vasodilation, can lead to the sensitization of pain‐sensitive neurons due to the activation of the nociceptor of the trigeminal system (Ruschel & De Jesus, 2021). Consequent to stimulation of the trigeminal ganglion, substance P, neurokinin, and calcitonin gene‐related peptide (CGRP) can be released (Matsuda et al., 2019). CGRP is shown to be involved in most physiological processes, such as dilating cerebral and dural blood vessels and releasing inflammatory mediators (Malhotra, 2016). The elevated levels of the mentioned neuropeptides are shown in the spinal fluid of individuals with chronic migraine (Anapindi et al., 2019; Riesco et al., 2017). Furthermore, according to available evidence, inflammatory factors such as interleukin (IL)‐1β, IL‐6, and tumor necrosis factor (TNF)‐α levels have also been shown to rise in patients with migraine, especially during migraine attack phases (Fidan et al., 2006; Martami et al., 2018). It is also believed that nitric oxide (NO) is a critical factor in migraine attack initiation by the NO/cyclic guanosine monophosphate (cGMP) pathway (Arzani et al., 2020; Capuano et al., 2009; Dodick, 2018). Evidence has reported a direct association between the dietary inflammatory index and migraine headaches (Ghoreishy et al., 2022). Consumption of fruits and vegetables is inversely associated with the dietary inflammatory index (Ghoreishy et al., 2022). Fruits and vegetables are good sources of anti‐oxidant components. Active components such as Indole‐3‐carbinol and sulforaphane in vegetables, including cabbage, broccoli, beets, parsley, spinach, and carrots, may act as CGRP antagonists and have been shown to be effective even more than administered drugs in some patients (Jain et al., 2015). Based on an in vivo study, grape seed extract treatment could decrease CGRP expression (Cady et al., 2010).

Furthermore, fruits, vegetables, beans, plant protein foods, and whole grains which may be protective against migraine headaches as shown by the current study, are rich in fiber. Fiber‐rich diets reduce inflammation by reducing the rate of glucose absorption, altering gut microflora, and leading to reduced production of inflammatory cytokines (Estruch et al., 2009). Fiber fermentation by the gut microbiota results in short‐chain fatty acid (SCFA) generation, including butyrate, propionate, and acetoacetate (Morrison & Preston, 2016). SCFAs can regulate gut and systemic immunity (Kim, 2021). Butyrate is involved in modulating regulatory T cell function, maintaining gut barrier integrity through the expression of tight junction proteins such as zonula occludens protein, occludin, and claudin‐2, and stabilizing hypoxia‐inducible factor (HIF), a vital factor for gut barrier integrity reserve and lower gut permeability to toxins (Chelakkot et al., 2018; Kespohl et al., 2017; Siddiqui & Cresci, 2021; Suzuki, 2020). Butyrate could also bypass portal circulation, reach the central nervous system (CNS), and generate neuroprotective effects (Noble et al., 2017).

Fruits, vegetables, and legumes are rich in magnesium, the mineral that is shown to be lower in plasma and the brain of migraine patients (Dibaba et al., 2014). Magnesium deficiency may cause cerebrovascular constriction, higher vascular reactivity, and a membrane receptor response to mediators, resulting in headache initiation (Mauskop & Altura, 1998). Magnesium plays a substantial role in the energy generation of mitochondria (Bianchi et al., 2004) and other physiological mechanisms affecting migraine pathophysiology, including vasoconstriction, platelet inhibition, and the secretion of serotonin (Gaul et al., 2015). Mitochondrial dysfunction is associated with migraine headaches (Lodi et al., 2001).

On the other hand, the current study has demonstrated that receiving more than 200 grams of fruit per 1000 calories, which is equivalent to 400 grams of fruit per 2000 calories, is associated with higher headache odds. As recommended by the World Health Organization, 400 grams of fruits and vegetables should be consumed daily (Organization WH, 2005, 2019). Despite the fact that some fruits, such as banana, watermelon, plum, grape, kiwi, raspberries, pineapple, and citrus fruits, like orange, are considered migraine triggers, it should be noted that migraine triggers may differ from patient to patient, and personal preferences may differ as well (Alpay et al., 2010; Silva‐Néto et al., 2021). Moreover, high consumption of fruits may act as an inflammatory factor. Fructose is a natural sugar in fruits. High intakes of dietary fructose are shown to be associated with inflammatory status and cause insulin resistance (Pereira et al., 2017). As shown by Shapiro et al., fructose consumption causes leptin resistance, which increases leptin levels despite the fat mass remaining unchanged. An increase in leptin levels leads to inflammation (Shapiro et al., 2008). A 50 g dose of fructose results in a spike in inflammatory markers like C‐reactive protein (CRP) about 30 min after consumption (Jameel et al., 2014).

The present study shows that consuming more than 138 g of “Dairy” per 1000 kcal is associated with higher odds of migraine headaches. This is equal to about more than one serving of dairy per 2000 kcal. High‐fat dairy contains saturated fats and may act as inflammatory factors (Lordan et al., 2018). Besides, in patients with cow's milk allergy, inflammatory immune responses might be triggered (Jo et al., 2014). Moreover, lactose intolerance patients may experience symptoms such as headaches when consuming high quantities of dairy products (Wilder‐Smith et al., 2018). A study on the association between migraines and lactose intolerance showed that 72% of the participants had lactose malabsorption, and more than half of them represented a substantial increase in headaches after lactose intake (Adokwe et al., 2019).

Additionally, we observed a direct association between “Total Protein Foods,” “Saturated Fats,” and “Sodium” intake and the odds of migraine headaches. Processed meats and fatty foods, rich in trans fatty acids and sodium, are reported as migraine‐triggering foods (Hoffmann & Recober, 2013). According to another study, migraine attack frequency is directly associated with western dietary patterns consisting of high consumption of sweetened drinks such as cola, salted nuts, processed meats, and fast food (Hindiyeh et al., 2020). Higher consumption of meat and a low intake of fruit and vegetables can lead to metabolic acidosis (Adeva & Souto, 2011), and a higher dietary acid load is directly associated with the odds of migraine headaches (Mousavi et al., 2021) and headache frequency (Lotfi et al., 2022). Reducing red and processed meat consumption may play an essential role in lowering migraine frequency (Altamura et al., 2020). A lower sodium intake is associated with a substantially lower risk of headaches (Amer et al., 2014).

It is interesting to note that we found an indirect association between migraine headaches and “Added Sugars” and “Refined Grains” in our study. A high‐fat or high‐sugar diet may trigger neuroinflammation through the microbiota‐gut‐brain axis and the TLR4 inflammatory pathway (Jamar et al., 2021). The Western dietary pattern, rich in simple sugar and saturated fatty acids and low in omega‐3 PUFAs and fiber, results in dysbiosis and gut microbiota diversity and quantity reduction and disturbs gut barrier function (Torres‐Fuentes et al., 2017). However, patients with migraine headaches may be addicted to simple sugar and find it difficult to reduce their consumption. Among the neurotransmitters affected by sugar is dopamine, which plays an important role in mood and, in addition, is a vital reward pathway in the brain, so it is necessary for pleasure and satisfaction. Consuming high amounts of sugar adversely affects dopamine regulation (Wiss et al., 2018). In such cases, when sugar is limited after a long period of time, patients may suffer from migraine‐type headaches (DiNicolantonio et al., 2018).

In such cases, when sugar is limited after a long period of time, patients may suffer from migraine‐type headaches. There is a possibility that some of these changes are due to diseases that are associated with migraine headaches.

### 4.1 The strengths and limitations of the study

The present study provides the first evidence of an association between HEI‐2015 and migraine odds. A validated FFQ was used for dietary data collection, and participants who reported energy intakes less than 600 or more than 4200 kcal were excluded. Incident migraine case selection could result in a causal interpretation and lower recall bias (Althubaiti, 2016; Thomas et al., 2013). Participation rates were high in both the migraine patients’ group and the controls. A specialist performed the diagnosis, and it was similar for both groups to control information bias. Besides, a trained dietician who was blinded to the diagnosis completed the interview. There are, however, several limitations to this study. Selection bias, measurement bias, and recall bias for FFQ may lead to misleading findings in a case–control study. In addition, alcohol and opium in Iran are taboos, so no data were collected on these variables. Moreover, while we examined adjusted models to account for potential confounding factors, it is important to acknowledge that certain baseline characteristics, such as age and BMI, differed between the case and control groups.

## 5 CONCLUSION

The present study showed that moderate adherence to the HEI‐2015 (with scores ranging from 65 to 76) reduces the odds of migraine headaches substantially. Additionally, consumption of “Total Vegetables,” “Greens and Beans,” and “Whole Grains” is associated with lower odds of migraine headaches. Alternatively, moderate fruit consumption has been found to lower migraine headache odds, while consuming too much fruit is associated with higher migraine headaches odds. There has been no consideration of a negative score for excessive consumption of protein foods, including animal protein, or excessive consumption of fruit and fruit juice in HEI‐2015. More studies are needed to confirm the findings revealed by the present study.

## AUTHOR CONTRIBUTIONS

**Danial Fotros:** Validation (equal); writing – original draft (equal); writing – review and editing (lead). **Morvarid Noormohammadi:** Validation (equal); writing – original draft (equal); writing – review and editing (supporting). **Mansoureh Togha:** Conceptualization (lead); project administration (equal). **Zeinab Ghorbani:** Data curation (equal); formal analysis (equal). **Azita Hekmatdoost:** Data curation (equal); formal analysis (equal). **Pegah Rafiee:** Data curation (equal); formal analysis (equal). **Zahra Torkan:** Data curation (equal); formal analysis (equal). **Pedram Shirani:** Data curation (equal); formal analysis (equal). **Hossein Ansari:** Data curation (equal); formal analysis (equal). **Ahmadreza Karami:** Data curation (equal); formal analysis (equal). **Faezeh Khorsha:** Data curation (equal); formal analysis (equal). **Soodeh Razeghi Jahromi:** Funding acquisition (lead); project administration (equal); supervision (lead).

## FUNDING INFORMATION

The Iranian Centre of Neurological Research, Neuroscience Institute, Grant Number 97–03–54‐39214 supported this study. The funding role included the design of the study, the collection, analysis, and interpretation of data, as well as the preparation of the manuscript. No funding was received for the publication of this article.

## CONFLICT OF INTEREST STATEMENT

D.F., M.N., S.RJ., M.T., Z.Gh., A.H., P.R., Z.T., P.Sh., H.A., A.K., and F.Kh. declare that they have no competing interests.

## Supporting information


Data S1.

## Data Availability

Datasets used and/or analyzed during this study can be obtained from the corresponding author upon reasonable request.
