# Supplementary material for: Healthy eating index 2015 might be associated with migraine headaches: Results from a Case–Control study
Source: Food Sci Nutr. 2024 Apr 21;12(7):5220–30. doi: 10.1002/fsn3.4168 (PMC11266926; doi:10.1002/fsn3.4168)
Supplement: Supplementary file 1 — Data S1. [file FSN3-12-5220-s001.docx]

| Supplementary Table 1. Healthy Eating Index-2015 components | | | |
| --- | --- | --- | --- |
| Components | Maximum Points | Standard for maximum point | Standard for minimum point (zero) |
| Total Fruits | 5 | >=0.8 cup equivalent per 1000 kilocalories | No Total Fruits |
| Whole Fruits | 5 | >=0.4 cup equivalent per 1000 kilocalories | No Whole Fruits |
| Total Vegetables | 5 | >=1.1 cup equivalent per 1000 kilocalories | No Total Vegetables |
| Greens and Beans | 5 | >=0.2 cup equivalent per 1000 kilocalories | No Greens and Beans |
| Whole grains | 10 | >=1.5 cup equivalent per 1000 kilocalories | No Whole grains |
| Dairy | 10 | >=1.3 cup equivalent per 1000 kilocalories | No Dairy |
| Total Protein Foods | 5 | >=2.5 cup equivalent per 1000 kilocalories | No Total Protein Foods |
| Seafood and Plant Proteins | 5 | >=0.8 cup equivalent per 1000 kilocalories | No Seafood and Plant Proteins |
| Fatty Acids | 10 | ((PUFA+MUFA)/SFA)>=2.5 | ((PUFA+MUFA)/SFA)=<1.2 |
| Refined Grains | 10 | =<1.8 ounce equivalent per 1000 kilocalories | >= 4.3 ounce equivalent per 1000 kilocalories |
| Sodium | 10 | =<1.1 grams per 1000 kilocalories | >= 2 grams per 1000 kilocalories |
| Added Sugars | 10 | =<6.5% of total calories intake | >= 26% of total calories intake |
| Saturated Fats | 10 | =<8% of total calories intake | >= 16% of total calories intake |
| Adapted from Krebs-Smith SM, Pannucci TE, Subar AF, Kirkpatrick SI, Lerman JL, Tooze JA, et al. Update of the Healthy Eating Index: HEI-2015. J Acad Nutr Diet. 2018;118(9):1591-602. | | | |

Danial Fotros^1^, Morvarid Noormohammadi^2,3^, Mansoureh Togha^4^, Zeinab Ghorbani^5^, Azita Hekmatdoost^1^, Pegah Rafiee^1,4^, Zahra Torkan^1,4^, Pedram Shirani^1,4^, Hossein Ansari^6^, Ahmadreza Karami^1,4^, Faezeh Khorsha^4^, Soodeh Razeghi Jahromi^1,7*^


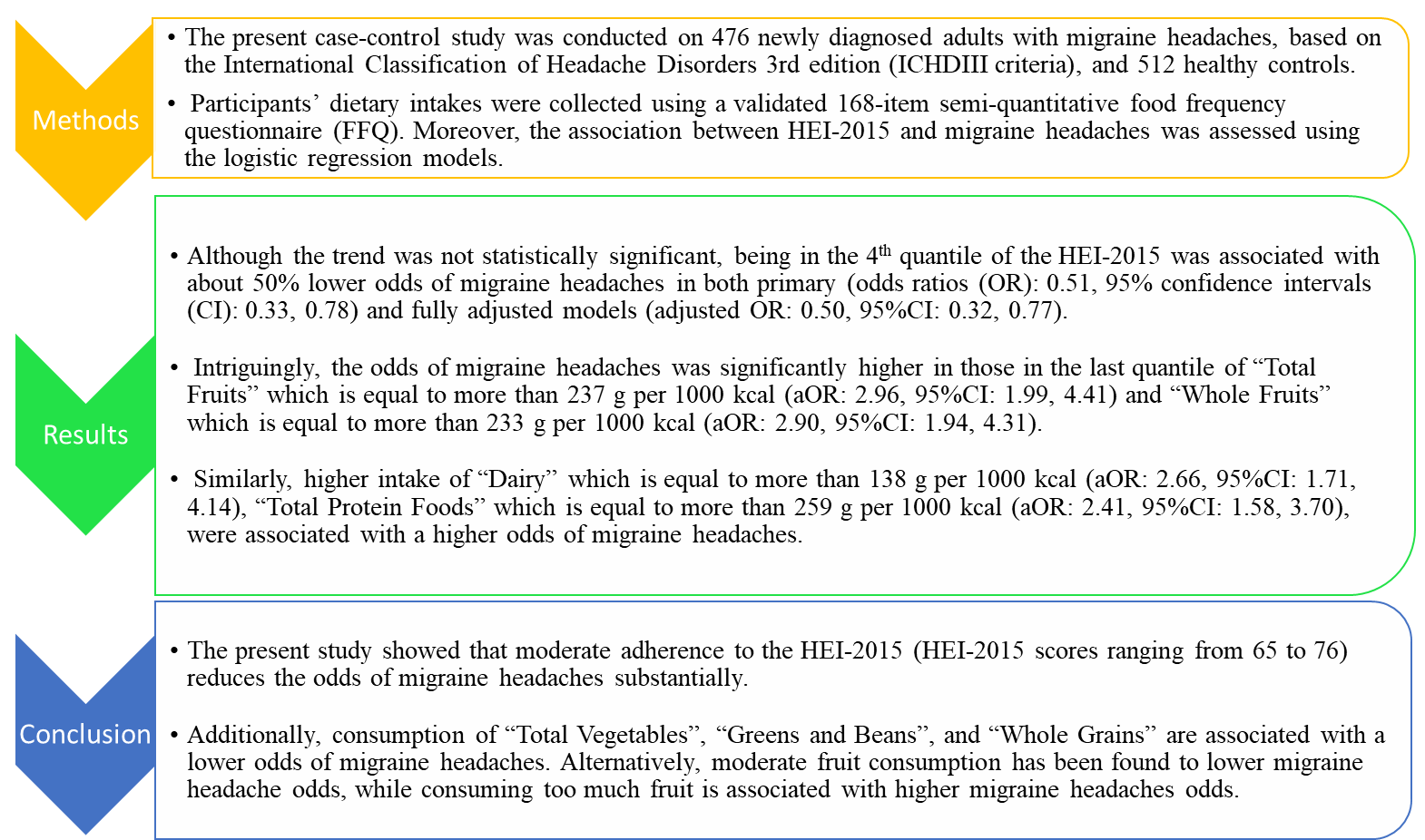


Figure 1. Graphical Table of Contents

This case-control study showed that moderate adherence to the HEI-2015 (HEI-2015 scores ranging from 65 to 76) reduces the odds of migraine headaches substantially. Additionally, consumption of “Total Vegetables”, “Greens and Beans”, and “Whole Grains” are associated with a lower odds of migraine headaches. Alternatively, moderate fruit consumption has been found to lower migraine headache odds, while consuming too much fruit is associated with higher migraine headaches odds.
